# Exploring the potential of *Crotalaria juncea* flower extracts as a source of antioxidants, antimicrobials, and cytoprotective agents for biomedical applications

**DOI:** 10.5114/bta.2023.132772

**Published:** 2023-12-21

**Authors:** Pawika Mahasawat, Sawai Boukaew, Poonsuk Prasertsan

**Affiliations:** 1 Programme in Biotechnology, Faculty of Science and Technology, Songkhla Rajabhat University, Songkhla, Thailand; 2 College of Innovation and Management, Songkhla Rajabhat University, Songkhla, Thailand; 3 Center of Excellence in Innovative Biotechnology for Sustainable Utilization of Bioresources, Faculty of Agro-Industry, Prince of Songkla University, Hatyai, Thailand

**Keywords:** antioxidant activity, antimicrobial activity, anti H_2_O_2_-induced cytotoxicity, *Crotalaria juncea* Linn, flower extract

## Abstract

Plants provide an unlimited source of bioactive compounds, possessing tremendous applications in the pharmaceutical industry. In the search for sources of antioxidants and antimicrobial agents against human pathogens, ethanol extracts of *Crotalaria juncea* flowers (CJ flower extract) were evaluated. The highest total phenolic (5.65 μg GAE/ml) and flavonoid (0.43 μg QE/ml) contents were observed in the 100 μg/ml CJ flower extract. To assess antioxidant activity, three *in vitro* antioxidant tests were employed: DPPH radical-scavenging, ABTS^+^ radical-scavenging, and hydroxyl radical-scavenging assay. The CJ flower extract demonstrated significant (*P* < 0.05) antioxidant activity, dependent on the percentage of solvent extraction and the specific assays utilized. The highest antioxidant activity was obtained with 100% ethanol extraction and using the hydroxyl radical-scavenging assay (56.63%). Antimicrobial activity was assessed against six human pathogens, including the fungi *Microsporum gypseum* and five Gram-positive bacteria (*Propionibacterium acnes*, *Staphylococcus aureus*, *Staphylococcus epidermidis*, *Streptococcus pyogenes*, and *Streptococcus mutans*), as well as one Gram-negative bacterium (*Escherichia coli* ). The CJ flower extract inhibited the growth of both fungal and bacterial pathogens. The cytotoxicity of the CJ flower extract was measured using a 3-(4,5-dimethylthiazol-2-yl)-2,5-diphenyltetrazolium bromide assay, and the highest concentration of the extract (100 μg/ml) did not affect L929 cell viability. Moreover, the CJ flower extract demonstrated the ability to suppress H_2_O_2_-induced toxicity in L929 cells. Overall, the CJ flower extract has potential as an alternative source for exploring new antioxidants, antimicrobial agents, and cytoprotectants that could prove valuable for biomedical applications.

## Introduction

Since ancient times, plant extracts have been used globally as a wide range of therapeutic agents. However, the rising incidence of antibiotic-resistant pathogens has propelled researchers to explore new antimicrobial agents, investigating the potential of plant extracts for drug development (Calixto et al., 2001; Rates, 2001; Gurib-Fakim, 2006). Considerable effort has been invested in exploring different natural sources to discover new antimicrobial compounds. The antimicrobial properties of plant products are widely recognized and have been utilized worldwide for developing novel drugs (Calixto et al., 2001; Rates, 2001; Gurib-Fakim, 2006). *Crotalaria juncea* Linn., cultivated worldwide, including in Thailand, encompasses a range of phytochemicals such as triterpenes, phenolics, flavonoids, alkaloids, amino acids, saponins, glycosides, tannins, and volatile oils, positioning it as a potential source of antioxidants and antimicrobial agents (Al-Snafi, 2016; Karl et al., 2020). Various *Crotalaria* species, including *C. juncea*, have demonstrated antimicrobial properties, establishing them as a promising group of plant products (Al-Snafi et al., 2016). Studies have found that extracts from various parts of Crotalaria species, including the leaves of *Crotalaria madurensis* and *Crotalaria capensis*, the root of *Crotalaria burhia*, and the seeds and flowers of *C. juncea* and *Crotalaria pallida*, exhibit antimicrobial activity against several bacteria, such as *Staphylococcus aureus, Enterococcus faecalis, Bacillus cereus, Bacillus subtilis, Escherichia coli, Pseudomonas aeruginosa*, and *Salmonella typhimurium* (Chouhan and Sushil, 2010; Sandeep et al., 2010; Dzoyem et al., 2014), as well as against fungal pathogens like *Fusarium oxysporum*, *Rhizoctonia solani*, and *Aspergillus fumigatus* (Pelegrini et al., 2009). Nonetheless, research into the antioxidant and antimicrobial properties of *C. juncea* L. flower extract remains limited. A sole study has reported that *C. juncea* L. seed extract can inhibit the growth of *E. coli*, *Klebsiella pneumonia*, *P. aeruginosa*, *S. aureus*, and *Vibrio cholerae* (Chouhan and Singh, 2010).

Phenolic compounds, secondary metabolites found in plants, not only serve various physiological functions within the plants themselves but also confer positive effects on human health by acting as antioxidants (Çaliskan and Polat, 2011). Antioxidants are critical in preventing pathogenic processes associated with various diseases such as cancer, cardiovascular disease, macular degeneration, cataracts, and asthma, and are also known to bolster immune function. These compounds protect the body against harmful free radicals generated during normal metabolism (Nakilcioglu and Hısıl, 2013). Beyond their antioxidative properties, various phenolic compounds derived from diverse plants – including pomegranate juice, *Artemisia campestris*, *Melilotus albus*, and *Dorycnium herbaceum* – have exhibited antimicrobial activity against various human pathogenic microorganisms (Turky2lmaz et al., 2013; Megdiche-Ksouri et al., 2015; Stefanovic et al., 2015).

At present, antioxidants are used as supplements to scavenge free radicals or activate defensive protein systems. Maintaining a proper redox balance is crucial for healthy cellular functions, as it supports the optimal functionality of redox-sensitive signalling proteins (Poljsak et al., 2013). HO-1, an oxidative stress response protein, catalyzes heme to produce biliverdin, iron, and carbon monoxide in equimolar amounts (Ines et al., 2012). Previous studies have shown that extracts from various plants, including *Koelreuteria henryi* Dummer flower (Tsai et al., 2020), *Quercus cerris* L. var. *cerris* L. (Şöhretoğlu et al., 2012), *Orostachys japonicus* A. Berger (Yoon et al., 2000), *Larrea divaricata* Cav. (Martino et al., 2014), and *Myrtus communis* (Ines et al., 2012), offer protection against hydrogen peroxide (H_2_O_2_)-induced oxidative damage or enzymatic oxidative stress across different cell lines.

To the best of our knowledge, information regarding the antimicrobial and antioxidant properties of *C. juncea* L. flower extract, particularly in Thailand, is limited. Consequently, this study was carried out to examine the effects of utilizing *C. juncea* L. flower extract, exploring its potential antimicrobial and antioxidant activities and anti-H_2_O_2_-induced toxicity in L929 cells.

## Materials and methods

### Microbial strains and cell culture

The antifungal and antimicrobial activities of *C. juncea* L. flower extract, herein referred to as CJ flower extract, were evaluated against the fungus *Microsporum gypseum* and six bacterial strains. Of these bacteria, five were Gram-positive: namely, *Propionibacterium acnes*, *S. aureus*, *Staphylococcus epidermidis*, *Streptococcus pyogenes*, and *Streptococcus mutans*, while *E. coli* was the lone Gram-negative strain. These microorganisms were obtained from the culture collection at the Thailand Institute of Scientific and Technological Research (TISTR) Phathumthani, Thailand, and the Laboratory of Microbiology at the Faculty of Sciences, Prince of Songkla University.

The L929 cells, a widely used mouse fibroblast cell line, served as the model system in our study to examine the effects of various treatments. Utilizing L929 cells enabled us to evaluate cellular responses and assess the potential therapeutic applications of the compounds tested in a controlled laboratory environment. The L929 cells were cultured in a medium composed of Dulbecco’s Modified Eagle’s Medium (obtained from Gibco, Paisley, UK), 10% fetal calf serum (obtained from Gibco, Paisley, UK), and penicillin (100 U/ml)/streptomycin (100 μg/ml) (obtained from Gibco, Paisley, UK). The cells were maintained in a humidified atmosphere of 5% CO_2_ at 37°C (Feizzadeh et al., 2008).

### Plant material and extraction procedures

The *C. juncea* (CJ) Linn. flowers were obtained from Lam Daeng, Singhanakhon District, Songkhla Province, in southern Thailand. After collection, the flowers were air-dried at 45°C for 48 h and ground into powder.

The powdered CJ flower (2 g) was combined with graded ethanol extracts (50 ml) at various concentrations (50, 70, and 100%). It was agitated on a rotary shaker at 120 rpm for 24 h. The residue from the flower powder extract was eliminated by filtering through Whatman filter paper no. 1. This extraction process was repeated two times. The filtrates were combined. The solvent was then evaporated under reduced pressure using a rotary evaporator to obtain a crude ethanol extract. This extract was stored in sealed vials at 4°C for further use. The percentage of extract yield (w/w) was computed based on the dry matter of the flower powder.

### Characteristics of the CJ flower extract

#### Total phenolic and flavonoid content

Total phenolic contents of each CJ flower extract were analyzed utilizing the Folin-Ciocalteu method. The CJ flower extracts were dissolved in ethanol at 10, 50, and 100 μg/ml (Fu et al., 2011). The total phenolic content was expressed as micrograms of gallic acid equivalents per milliliter (μg GAE/ml), utilizing a calibration curve of gallic acid with a linearity range from 0 to 50 μg/ml (*R* ^2^ > 0.99) (Ainsworth and Gillespie, 2007). All experiments were performed in triplicate.

In terms of total flavonoid contents, each CJ flower extract was determined using an aluminum chloride assay. For this analysis, the CJ flower extracts were dissolved in ethanol at a concentration of 100 μg/ml (Koolen et al., 2013). The total flavonoid contents were then expressed as micrograms of quercetin equivalents per milliliter (μg QE/ml), based on the calibration curve of quercetin, which exhibited a linearity range from 0 to 100 μg/ml (*R* ^2^ > 0.99) (Chang et al., 2002). These experiments, too, were performed in triplicate.

#### Cytotoxicity of CJ flower extract

The cytotoxicity of CJ flower extract on the L929 cell line was evaluated through a modified 3-(4,5-dimethylthiazol-2-yl)-2,5-diphenyltetrazolium bromide (MTT) assay, adhering to the method outlined by Hamid et al. (2012). Initially, L929 cells were seeded into a 96-well plate at a concentration of 1 × 10^4^ cells and incubated for 24 h. Subsequently, the cell culture medium was supplemented with CJ flower extract at varying concentrations (0, 1, 5, 10, 50, and 100 μg/ml), followed by another 24 h of incubation. Postincubation, the cells were treated with MTT solution for 4 h, after which the formazan crystal was dissolved using dimethyl sulfoxide (DMSO). All experiments were conducted in triplicate. The absorbance of the solution was measured at 562 nm using a microplate reader (EZ Read 800 Plus Research, Biochrom, England). The cell viability was calculated using the following formula:


$$\text{Cell viability}\left[\%\text{ of control}\right]=\frac{A_{562\text{ nm}}\text{ of treated cells}}{A_{562\text{ nm}}\text{ of control cells}}\times 100$$

### Bioactivity of CJ flower extract

#### Antioxidant activity

The antioxidant activity of the CJ flower extract was evaluated through the DPPH radical-scavenging assay, ABTS^+^ radical-scavenging activity, and hydroxyl radicalscavenging assay, using the methods outlined by Ballard et al. (2010), Yusoff et al. (2015), and Chen et al. (2018), respectively.

For the DPPH radical-scavenging assay, CJ flower extract at a concentration of 100 μg/ml was prepared, and a 0.1 mM DPPH radical solution in methanol was added. The mixture was incubated in the dark at room temperature for 30 min. Absorbance was measured at 517 nm using a UV-visible spectrophotometer. The equation to calculate the percentage of DPPH radical scavenging activity was as follows:


$$\text{DPPH radical inhibition}\left[\%\right]=\frac{A_{c}-A_{s}}{A_{c}}\times 100$$

where *A*_c_ and *A*_s_ represented the absorbance of the control and the sample, respectively.

For the ABTS^+^ radical-scavenging activity, 10 μl of CJ flower extract at 100 μg/ml was added to 1 ml of ABTS^+^ radical solution. After incubation in the dark for 6 min at room temperature, the absorbance was measured at 734 nm using a UV-visible spectrophotometer. The test was conducted in triplicate. The percentage of ABTS^+^ radical scavenged was determined as follows:


$${\text{ABTS}}^{\text{+}}\text{ radical inhibition}\left[\%\right]=\frac{A_{c}-A_{s}}{A_{c}}\times 100$$

where *A*_c_ and *A*_s_ denoted the absorbance of the control and the sample, respectively.

For the hydroxyl radical-scavenging assay, a mixture comprising 200 μl of FeCl_3_ (200 μM) and ethylene diamine tetraacetic acid (EDTA) (1.04 mM) at a 1 : 1 ratio, 100 μl of 2-deoxy-D-ribose (28 mM), 100 μl of hydrogen peroxide (H O ; 1 mM), and 100 μl of ascorbic acid (1 mM) was added to 500 μl of CJ flower extract solution at a concentration of 100 μg/ml and incubated at 37°C for 1 h. The resulting mixture was then combined with 1 ml of thiobarbituric acid (TBA) (1% w/v) and trichloroacetic acid (TCA) (2.8% w/v), and it was subsequently incubated at 80°C for 20 min. The absorbance of the final solution was measured at 532 nm, and the scavenging activity was determined using the following equation:


$$\text{OH radical inhibition}\left[\%\right]=\frac{A_{c}-A_{s}}{A_{c}}\times 100$$

where *A*_c_ and *A*_s_ denoted the absorbance of the control and the sample, respectively.

#### Antimicrobial activity of the CJ flower extract

To test the antimicrobial activity of the CJ flower extract against bacterial and fungal pathogens, the methodology elucidated by Awouafack et al. (2013) was employed, utilizing the microdilution method to determine the minimum inhibitory concentration (MIC), minimum fungicidal concentration (MFC), and minimum bactericidal concentration (MBC). Crude extracts were tested across two-fold serial dilutions ranging from 0.25 to 500 μg/ml.

The evaluation of the antibacterial activity of CJ flower extract was tested using a previously described methodology by Awouafack et al. (2013). In brief, 10 μg/ml of the CJ flower extract, dissolved in dimethyl sulfoxide (DMSO), was serially diluted two-fold with sterile distilled water in 96-well microtiter plates. Subsequently, 100 μl of bacterial culture was introduced to each well. To prevent contamination, the plates were sealed in plastic bags and incubated under conditions specific to each bacterial pathogen. After incubation, INT was added to each well, and the plates underwent additional incubation. The MIC was identified as the lowest concentration inhibiting microbial growth.

Utilizing the aforementioned microdilution method, the MFC and MBC were determined using two-fold serial dilutions of crude extracts, again ranging from 0.25 to 500 μg/ml. Bacterial cultures, with densities of 1–2 × 10^8^ colony-forming units (CFU) per ml, were added to 99 ml of varying growth media. One milliliter of each bacterial pathogen was taken from freshly prepared overnight bacterial cultures in medium broth, employing DMSO as a negative control. The experiment was conducted in triplicate.

To assess the antifungal activity of the CJ flower extract, the extract was dissolved in DMSO and serially diluted in a 96-well microplate. Each well was then inoculated with 100 μl of *M. gypseum* (8 × 10^3^ spores ml) culture in sabouraud dextrose broth (SDB), and the microplate was incubated at 25°C. The fungal growth was indicated by the addition of INT to each well, with the MIC value being recorded as the lowest concentration of the sample that inhibited the fungal growth. Clotrimazole (obtained from Sigma-Aldrich, St Louis, MO, USA) and DMSO were used as a positive and negative control, respectively. The reduction of the color intensity of the formazan product indicated fungal growth inhibition, with the MIC being identified as the lowest concentration that manifested this reduction. This method, previously described by Masoko et al. (2005), involves the reduction of the colorless salt of tetrazolium to a red-colored formazan product by biologically active organisms. The experiment was performed in triplicate.

#### Efficacy of the CJ flower extract to protect L929 cells after stimulated by H_2_O_2_

This study investigated the ability of the CJ flower extract to protect L929 cells from H_2_O_2_-induced cell damage. L929 cells were seeded in a 96-well plate at a density of 1 × 10^4^ cells/well. According to the protocol delineated by Vanlangenakke et al. (2011), the cells were treated with 50 μl of CJ flower extract at varying concentrations (1, 5, 10, 50, and 100 μg/ml) alongside 50 μl of H_2_O_2_ (250 μM) for a 24-h duration. Subsequently, cell viability was assessed using the MTT assay, and the cell viability was calculated based on the absorbance, as outlined by Lai et al. (2018). The experiment was performed in triplicate.

### Statistical analysis

Data were analyzed using either a one-way or two-way analysis of variance (ANOVA) with the Statistical Package for the Social Sciences (SPSS) version 26 for Windows. When necessary, means were evaluated utilizing Duncan’s multiple range test (DMRT), considering significance at *P* < 0.05.

## Results

### Characteristics of the CJ flower extract

#### Extract yield of CJ flower extracted with different ethanol concentrations

The yield of CJ flower extracts varied between 15.50 and 16.13% (Table 1). The use of varying ethanol concentrations (50, 70, and 100%) for extract preparation did not significantly (*P* > 0.05) affect the total yield of crude extracts from *C. juncea* L. According to the results, the highest yield was obtained using 50% ethanol (16.13%), followed by 70% ethanol (16.10%), and lastly, 100% ethanol (15.50%).

**Table 1:** Characteristics and crude yield of CJ flower extracts obtained using different ethanol concentrations

Ethanol concentration [v/v]	Yield of extracts [g]	Yield of extracts [%]	CJ flower extract color
50%	0.323 ^a^	16.13 ^a^	black
70%	0.322 ^a^	16.10 ^a^	black
100%	0.310 ^a^	15.50 ^a^	yellow

#### Total phenolic and flavonoid content

**Fig. 1:**
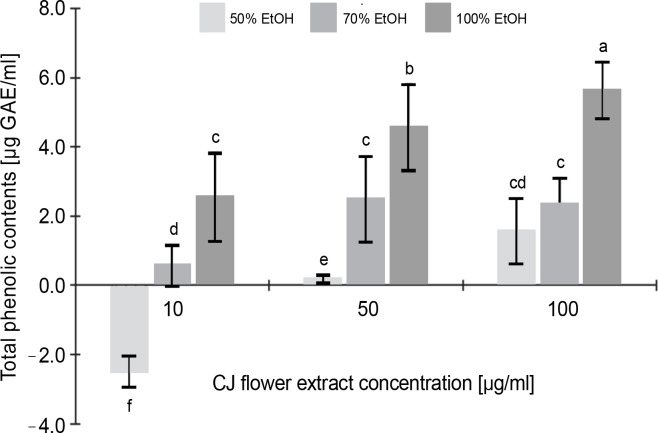
Total phenolic contents (as gallic acids equivalents) of *C. juncea* L. flower extract (CJ flower extract at a concentration of 10, 50, 100 μg/ml) obtained using different ethanol concentrations of 50, 70, and 100%; absorbance values represent triplicates of different samples analyzed; values with the same letter are not significantly different (*P* < 0.05) between samples

Figure 1 illustrates the total phenolic content of the CJ flower extract, highlighting a noteworthy variation (*P* < 0.05) contingent upon both the CJ flower extract concentration (10, 50, and 100 μg/ml) and the ethanol extraction concentration (50, 70, and 100%). A significant increase (*P* < 0.05) in phenolic content was observed with the elevation of ethanol extraction percentage, ranging from 1.55 to 5.63 μg GAE/ml (at a CJ flower extract concentration of 100 μg/ml). The greatest total phenolic content, 5.65 μg GAE/ml, was noted in the CJ flower extract obtained through 100% ethanol extraction.

**Fig. 2:**
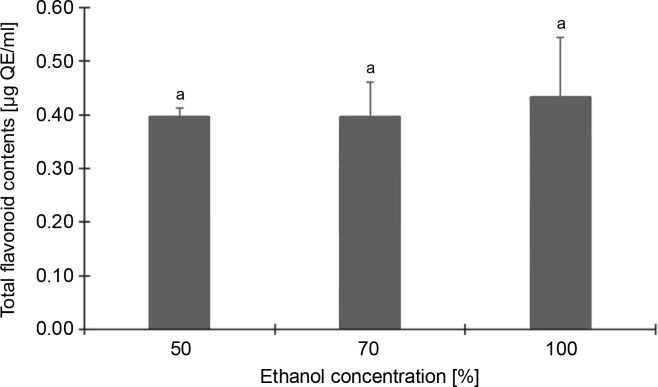
Total flavonoid contents (as quercetin equivalents) of *C. juncea* L. flower extract (at 100 μg/ml) at different ethanol concentrations of 50, 70, and 100%; absorbance values represent triplicates of different samples analyzed; values with the same letter are not significantly different (*P* < 0.05) between ethanol concentrations

Conversely, Figure 2, which depicts the total flavonoid content of the CJ flower extract at varying ethanol concentrations (50, 70, and 100%), reveals no significant (*P* > 0.05) differences in flavonoid content across different ethanol extraction concentrations. The highest total flavonoid content was identified in the 100% ethanol extraction, registering 0.43 μg QE/ml, followed by 50% (0.40 μg QE/ml) and 70% (0.40 μg QE/ml) ethanol extractions.

#### Cytotoxicity of CJ flower extract on L929 cells

**Fig. 3:**
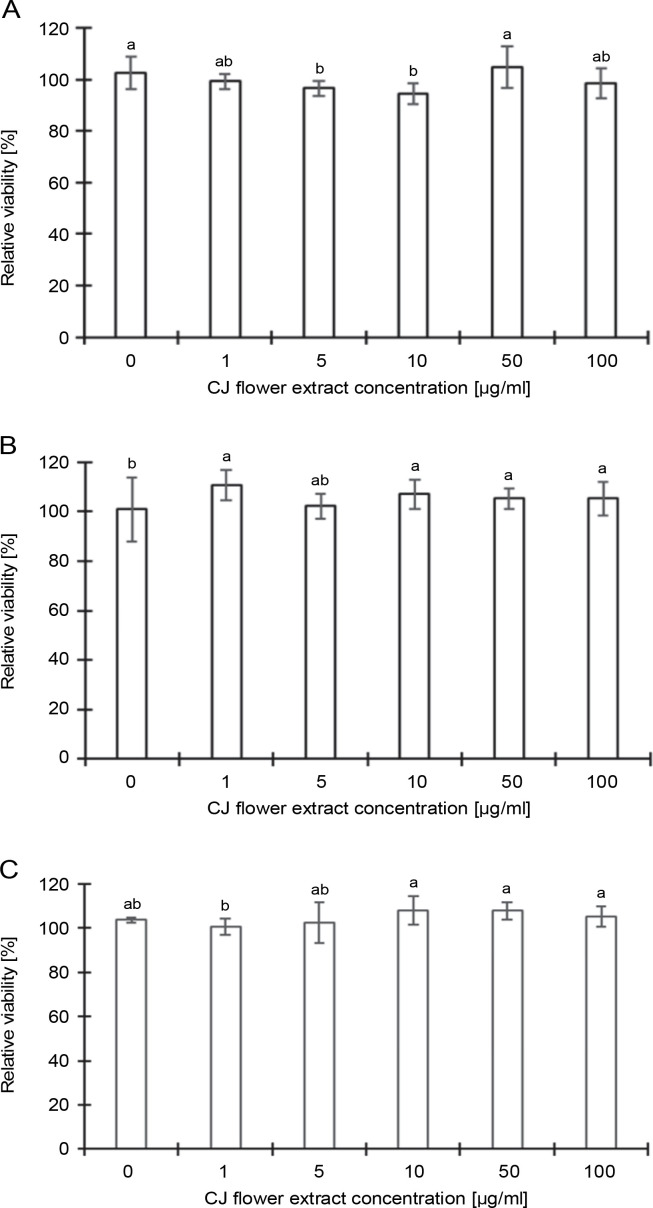
Toxicity of CJ flower extract at different concentrations (0, 1, 5, 10, 50, and 100 μg/ml) on a relative number of L929 cells by a 3-(4,5-dimethylthiazol-2-yl)-2,5-diphenyltetrazolium bromide (MTT) assay; (A) 50% ethanol extraction, (B) 70% ethanol extraction, and (C) 100% ethanol extraction; absorbance values represent triplicates of different samples analyzed; values with the same letter are not significantly different (*P* < 0.05) between CJ flower extract concentrations

Figure 3 illustrates the impact of different concentrations (0, 1, 5, 10, 50, and 100 μg/ml) of CJ flower extracts on the relative number of L929 cells, as assessed by the MTT assay. Results indicate that a 4 h treatment with CJ flower extract did not present evident adverse effects on L929 cells at concentrations from 1 to 100 μg/ml compared to the control group (0 μg/ml). Furthermore, no significant (*P* > 0.05) reduction in cell viability was observed upon exposure to CJ flower extract at the tested ethanol concentrations (50, 70, and 100%). Thus, the findings imply that the CJ flower extract exerts no discernible effect on L929 cells.

### Bioactivity of CJ flower extract

#### Antioxidant activity

**Fig. 4:**
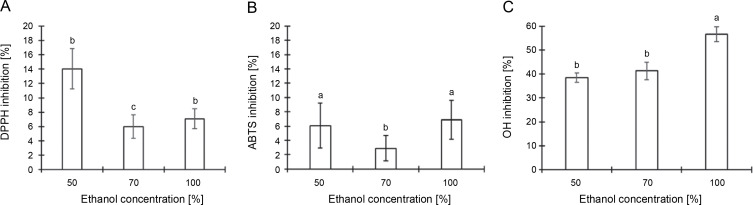
Antioxidant activity of *C. juncea* L. flower extract (at 100 μl/ml) at different ethanol concentrations of 50, 70, and 100% and three different assays; DPPH radical scavenging activity (A), ABTS radical scavenging activity (B), and hydroxyl radical scavenging assay (C); absorbance values represent triplicates of different samples analyzed; values with the same letter are not significantly different (*P* < 0.05) between ethanol concentrations

Figure 4 details the results from an analysis of the total antioxidant activity of CJ flower extract, utilizing various ethanol concentrations (50, 70, and 100%) across three distinct assays. The DPPH radical-scavenging assay revealed that the maximal anti-DPPH radical activity, 14.05%, was attained with a 50% ethanol extraction, followed by 7.08% with 100% ethanol and 5.98% with 70% ethanol extraction (Fig. 4A). According to Figure 4B, the scavenging impacts on the ABTS^+^ radical followed the sequence: 100% ethanol (6.91%) > 50% ethanol (6.07%) > 70% ethanol (2.89%). The hydroxyl radical-scavenging activity, measured via the inhibition of 2-deoxyribose degradation by free radicals generated by the Fenton reaction, demonstrated that the scavenging activity of the CJ flower extract adhered to the order: 100% ethanol (56.63%) > 70% ethanol (41.27%) > 50% ethanol (38.49%) (Fig. 4C). Such findings intimate that the antioxidant activity of the CJ flower extract is modulated by both the percentage of ethanol used in the extraction and the specific assay employed.

#### Antimicrobial activity

Utilizing the microdilution method, the impact of CJ flower extract, obtained through 100% ethanol extraction and ranging in concentrations from 0.25 to 500 μg/ml, was tested against an array of microbial pathogens. These included *P. acnes*, *S. aureus*, *S. epidermidis*, *S. pyogenes*, *S. mutans* (all Gram-positive bacteria), *E. coli* (a Gram-negative bacteria), and *M. gypseum* SHMU-4 (a fungal species). Comprehensive results, encompassing both MIC and MBC/MFC values, are detailed in Table 2.

**Table 2:** MIC and MBC/MFC activities against pathogenic microorganisms

S. No.	Name of the organisms	MIC (μg/ml)	MBC/MFC (μg/ml)
1	*P. acnes*	200	> 500
2	*S. aureus*	128	500
3	*S. epidermidis*	64	200
4	*S. pyogenes*	16	128
5	*S. mutans*	64	200
6	*E. coli*	500	> 500
7	*M. gypseum*	200	500

Notably, the CJ flower extract demonstrated potent activity against Gram-positive bacteria, exhibiting MIC values between 16 and 200 μg/ml, which was higher than that for Gram-negative bacteria, with a MIC value of 500 μg/ml. The lowest MIC and MBC/MFC values were observed against *S. pyogenes* (16 μg/ml) and *M. gypseum* (200 μg/ml), respectively.

Interestingly, the extract demonstrated a more pronounced effect against Gram-positive bacteria than Gram-negative bacteria. This variance in susceptibility may be attributed to divergent cell wall structures inherent to the different bacterial types. Specifically, the thicker peptidoglycan layer in Gram-positive bacteria’s cell walls may present a target for the extract’s bioactive compounds. Conversely, Gram-negative bacteria, which boast an additional outer membrane alongside a thinner peptidoglycan layer, could potentially restrict the extract’s bioactive components’ accessibility due to this added barrier. Furthermore, the MBC/MFC values underscore that the CJ flower extract exhibited inhibitory actions against bacterial and fungal pathogens.

#### CJ flower extract suppresses H_2_O_2_-stimulated toxicity in L929 cells

**Fig. 5:**
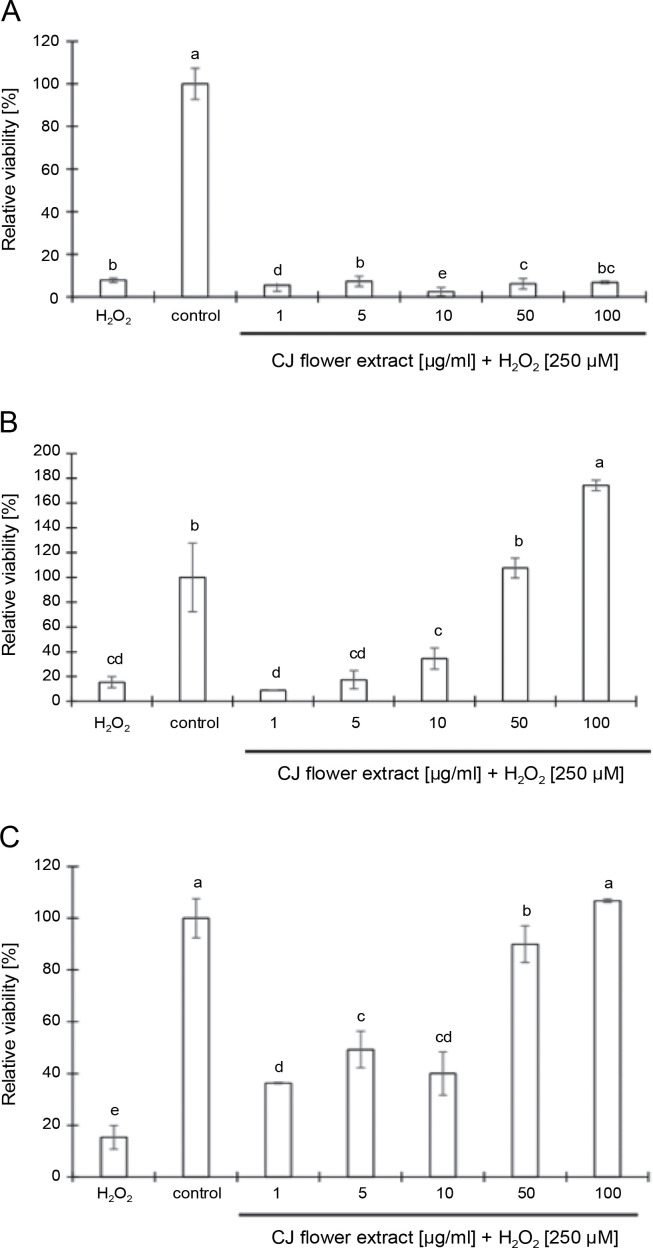
Efficacy of C. *juncea L*. flower extract (at a concentration of 1 to 100 μg/ml) by different ethanol extraction of 50% (A), 70% (B), and 100% (C) to protect L929 cells after stimulated by H_2_O_2_ (250 μM) using a 3-(4,5-dimethylthiazol-2-yl)-2,5-diphenyltetrazolium bromide (MTT) assay; the negative control involved L929 cells without the addition of CJ flower extract and H_2_O_2_; in the positive control, only H_2_O_2_ was added to the L929 cells without the presence of CJ flower extract; absorbance values represent triplicates of different samples analyzed

The study investigated the ability of CJ flower extract, extracted using different concentrations of ethanol (50, 70, and 100%) and applied at concentrations ranging from 1 to 100 μg/ml, to protect L929 cells against H_2_O_2_-induced cytotoxicity. Remarkably, the extract obtained from 50% ethanol extraction did not show anti-oxidative effects against H_2_O_2_-induced cytotoxicity, even at the highest concentration of 100 μg/ml (Fig. 5A). Conversely, an intriguing observation emerged when increasing concentrations of CJ flower extract from 1 to 100 μg/ml, which evidenced a significant (*P* < 0.05) and dose-dependent reduction of H_2_O_2_-induced toxicity in L929 cells (Fig. 5B and Fig. 5C). Notably, a concentration of 100 μg/ml of CJ flower extract bolstered a higher survival rate in L929 cells relative to lower concentrations.

## Discussion

Various plant extracts, such as Fagonia indica, Ephedra intermedia, Cissus arnotiana, Vernonia cinerea, Coronopus didymus, Ocimum basilicum L., Ziziphus lotus L., Berberis aristata, Allium ampeloprasum L., and Zingiber officinale Rosc. are recognized as sources of antioxidants and antimicrobial agents (Alara et al., 2018; Ahmed et al., 2019; Khaliq et al., 2019; Khoshnamvand et al., 2019; Rajeshkumar et al., 2019). However, limited knowledge is available on the antimicrobial and antioxidant activities of the ethanol extract of C. juncea L. flower. This research presents updated information on the antioxidant and antimicrobial activities and anti H O induced cytotoxicity of the ethanol extract of C. juncea L. flower (CJ flower extract).

Pivotal to our exploration is the role of phenolic compounds, distinguished for their antioxidant properties and contributions to health, pervading numerous medicinal plants and, by extension, our diets. Our study fortifies that CJ flower extract, when subjected to 100% ethanol extraction at a concentration of 100 μg/ml, unveils optimal levels of phenolic and flavonoid contents. This aligns with findings noted by Dirar et al. (2019) in their exploration of Blepharis linariifolia Pers. A crosscomparison with other plant extracts, such as those from Thymus species (Tohidi et al., 2017), Zingiber officinale Rosc. (Ali et al., 2018), Oliveria decumbens Vent. (Esmaeili et al., 2018), Citrus reticulata Blanco (Hua et al., 2018), Olea europaea (Şahin et al., 2018), and Blepharis linariifolia (Dirar et al., 2019), was reported. The phenolic contents of ethanol extracts varied significantly, influenced by the nature and quantity of secondary plant metabolites used.

The exploration of phenolic compounds and flavonoids is well-acknowledged for their capacity to neutralize free radicals and serve as antioxidants. To rapidly screen substances and predict their in vivo activity, various in vitro techniques have been employed to determine their antioxidant activity (Nunes et al., 2012; Saeed et al., 2012). In this specific research domain, a meticulous examination of the antioxidant activities of CJ flower extracts was conducted, benchmarking at a concentration of 100 μg/ml and utilizing three distinct ethanol concentrations (50, 70, and 100%) as part of the investigation mechanism. Three assays were utilized in this research to gauge the antioxidant activities: the DPPH radical-scavenging assay, ABTS^+^ radical-scavenging activity, and the hydroxyl radical-scavenging assay. Intriguingly, the hydroxyl radical-scavenging assay revealed that the extract, when subjected to 100% ethanol extraction, displayed the highest antioxidant activity against the other fractions under examination. In contrast, no significant differences in antioxidant activity were observed between the DPPH radical-scavenging and ABTS^+^ radical-scavenging assays, which did not reveal substantial disparities in antioxidant activity. It was discerned that the influence of ethanol concentrations on detected antioxidant activity varied depending on the assay technique employed. The choice of ethanol concentration during the extraction process emerged as a pivotal factor, markedly impacting extraction efficiency and, consequently, the antioxidant potential revealed in the results. Given that antioxidant activity assessment techniques, such as DPPH and ABTS^+^ radical-scavenging assays, target distinct reactive oxygen species (ROS) and are anchored in varied mechanisms, the resultant antioxidant activity can be significantly influenced by the interplay between ethanol concentration and the specific ROS targeted by each technique. Future studies should investigate the relationship between ethanol concentration, extraction efficiency, and antioxidant activity. Such research would be integral to optimizing extraction conditions, thereby amplifying antioxidant properties. The results suggest from the current study propagate the notion that CJ flower extracts are rich in phytochemical constituents, bestowing them with the potential to scavenge free radicals and thereby attenuate oxidative damage.

Various bacteria, including antibiotic-resistant strains and fungal species, have been found susceptible to the antimicrobial effects of plant extracts (de Oliveira et al., 2017; Yuan and Yuk, 2018; Sabo and Knezevic, 2019). Our study demonstrated that CJ flower extracts exhibited higher efficacy in inhibiting Gram-positive bacteria, with an MIC range of 16–200 μg/ml, compared to Gram-negative bacteria, with an MIC of 500 μg/ml. This result aligns with previous findings on Ficus carica L. (Olufemi et al., 2013; Mahmoudi et al., 2016). The reason for the lower susceptibility of Gram-negative bacteria is not yet fully understood, but it could be attributed to their outer membrane, which contains peptidoglycans and lipopolysaccharides and acts as a strong permeability barrier due to its hydrophilic nature (Nourbakhsh et al., 2020). Additionally, the CJ flower extracts exhibited strong antifungal activity against M. gypseum, with a MIC value of 200 μg/ml. Overall, these antimicrobial results suggest that CJ flower extracts have potential biomedical applications, as they can effectively inhibit a broad range of human-pathogenic bacteria and fungi.

In biological systems, the hydroxyl radical stands out as a highly reactive oxygen species, capable of damaging cells by interacting with polyunsaturated fatty acids within cell membrane phospholipids (Halliwell et al., 1981; Khan et al., 2012; Saeed et al., 2012). Hydroxyl radicals can be generated through the interaction of ferrous ions with H_2_O_2_, which may subsequently decompose into oxygen and water, concurrently generating hydroxyl radicals (˙OH) and leading to lipid peroxidation and DNA damage (Sahreen et al., 2011; Saeed et al., 2012). This study aimed to evaluate the potential of CJ flower extracts, extracted using varying ethanol concentrations (50, 70, and 100%) and at concentrations ranging from 1 to 100 μg/ml, to suppress H O –induced toxicity in L929 cells. Our results revealed a notable dose-dependent reduction of H_2_O_2_-induced cytotoxicity upon treatment with CJ flower extracts, underscoring their potential as effective agents in attenuating oxidative damage. Further research is warranted to elucidate the molecular mechanisms underlying these observed effects and to identify the specific bioactive compounds responsible for the anti-oxidative properties of CJ flower extracts. Interestingly, the CJ flower extracts obtained using 70 and 100% ethanol at a concentration of 100 μg/ml exhibited heightened efficacy in suppressing H_2_O_2_-induced cell damage without impacting L929 cell viability. Hence, our study suggests that CJ flower extracts could be a potential protective agent against H_2_O_2_-induced oxidative damage.

The insights from this study spotlight CJ flower extracts as a promising ingredient in antioxidant agents, owing to their ability to effectively suppress H_2_O_2_-induced cell damage without compromising L929 cell viability. However, it remains imperative to consider the cellular protective effects of extracts from other plants to more comprehensively understand comparative efficacy. Several studies have investigated the cytoprotective effects of various plant extracts, such as mangosteen (Sattayasai1et al., 2013), millet, rice, wheat, brown rice, oat, buckwheat, corn, and adlay (Zhao et al., 2017), unveiling that these extracts manifest cytoprotective effects through different mechanisms, including the suppression of ROS levels and caspase-3 activity, and the activation of SOD, CAT and GSH-Px activities.

## Conclusion

In conclusion, CJ flower extracts have demonstrated a significant amount of polyphenols and flavonoids, which contribute to the health-promoting properties observed. The extracts exhibit promising effects, including antioxidant properties, antimicrobial activities against a wide range of bacteria and fungi, and anti H O –activated cell damage. However, to further improve their antioxidant, antimicrobial, and cytoprotective potential, identification and purification of specific phenolic compounds present in the extracts are requisite. Future studies should focus on isolating these compounds to enhance their effectiveness as natural antioxidants, antimicrobials, and cytoprotective agents, thereby exploring new avenues for their application across various fields.

## Data Availability

Not applicable.

## References

[cit0001] Ahmed A.F., Attia F.A.K., Liu Z., Li, C., Wei J., Kang W. (2019) Antioxidant activity and total phenolic content of essential oils and extracts of sweet basil (Ocimum basilicum L.) plants. Food Sci. Hum. Well. 8: 299–305.

[cit0002] Ainsworth E.A., Gillespie K.M. (2007) Estimation of total phenolic content and other oxidation substrates in plant tissues using Folin-Ciocalteu reagent. Nat. Protoc. 2: 875–877. 17446889 10.1038/nprot.2007.102

[cit0003] Alara O.R., Abdurahman N.H., Ukaegbu C.I. (2018) Soxhlet extraction of phenolic compounds from Vernonia cinerea leaves and its antioxidant activity. J. Appl. Res. Med. Aromat Plants 11: 12–17.

[cit0004] Ali A.M.A., El-Nour M.E.M., Yagi S.Y. (2018) Total phenolic and flavonoid contents and antioxidant activity of ginger (Zingiber officinale Rosc.) rhizome, callus and callus treated with some elicitors. J. Genet. Eng. Biotechnol. 16: 677–682. 30733788 10.1016/j.jgeb.2018.03.003 PMC6353720

[cit0005] Al-Snafi A.E. (2016) The contents and pharmacology of Crotalaria juncea – a review. IOSR J. Pharm. 6: 77–86.

[cit0006] Awouafack M.D., McGaw L.J., Gottfried S., Mbouangouere R., Tane P., Spiteller M., Elof J.N. (2013) Antimicrobial activity and cytotoxicity of the ethanol extract, fractions and eight compounds isolated from Eriosema robustum (Fabaceae). BMC Complement. Altern. Med. 13: 289. 24165199 10.1186/1472-6882-13-289 PMC3817817

[cit0007] Ballard T.S., Mallikarjunan P., Zhou K., O’Keefe S. (2010) Microwave-assisted extraction of phenolic antioxidant compounds from peanut skins. Food Chem. 120: 185–1192. 10.1021/jf8030925 19284759

[cit0008] Çaliskan O., Polat A.A. (2011) Phytochemical and antioxidant properties of selected fig (Ficus carica L.) accessions from the eastern Mediterranean region of Turkey. Sci. Hortic. 128: 473–478.

[cit0009] Calixto J.B., Scheidt C., Otuki M., Santos A.R.S. (2001) Biological activity plant extract: novel analgesic drugs. Expert Opin. Emerg. Drugs 6: 261–279. 15989526 10.1517/14728214.6.2.261

[cit0010] Chang C.C., Yang M.H., Wen H.M., Chern J.C. (2002) Estimation of total flavonoid content in propolis by two complementary colorimetric methods. J. Food Drug. Anal. 10: 178–182.

[cit0011] Chen Y., Liu X., Wu L., Tong A., Zhao L., Liu B., Zhao C. (2018) Physicochemical characterization of polysaccharides from Chlorella pyrenoidosa and its anti-ageing effects in Drosophila melanogaster. Carbohydr. Polym. 185: 120–126. 29421048 10.1016/j.carbpol.2017.12.077

[cit0012] Chouhan H., Sushil K.S. (2010) Antibacterial activity of seed and flower parts of Crotalaria juncea Linn. Am.-Euras. J. Sci. Res. 5: 212–215.

[cit0013] Dirar A.I., Alsaadi D.H.M., Wada M., Mohamed M.A., Watanabe T., Devkota H.P. (2019) Effects of extraction solvents on total phenolic and flavonoid contents and biological activities of extracts from Sudanese medicinal plants. S. Afr. J. Bot. 120: 261–267.

[cit0014] Dzoyem J.P., Mc Gaw L.J., Eloff J.N. (2014) In vitro antibacterial, antioxidant and cytotoxic activity of acetone leaf extracts of nine under investigated Fabaceae tree species leads to potentially useful extracts in animal health and productivity. BMC Complement. Altern. Med. 14: 1–7. 24885143 10.1186/1472-6882-14-147 PMC4032570

[cit0015] Esmaeili H., Karami A., Maggi F. (2018) Essential oil composition, total phenolic and flavonoids contents, and antioxidant activity of Oliveria decumbens Vent. (Apiaceae) at different phenological stages. J. Clean Prod. 198: 91–95.

[cit0016] Feizzadeh B., Afshari J.T., Rakhshandeh H., Rahimi A., Brook A., Doosti H. (2008) Cytotoxic effect of saffron stigma aqueous extract on human transitional cell carcinoma and mouse fibroblast. Urol. J. 5: 161–167. 18825622

[cit0017] Fu L., Xu B.T., Gan R.Y., Zhang Y., Xu X.R., Xia E.Q., Li H.B. (2011) Total phenolic contents and antioxidant capacities of herbal and tea infusions. Int. J. Mol. Sci. 12: 2112–2124. 21731430 10.3390/ijms12042112 PMC3127106

[cit0018] Gurib-Fakim A. (2006) Medicinal plants: traditions of yesterday and drugs of tomorrow. Mol. Aspects Med. 27: 1–93. 16105678 10.1016/j.mam.2005.07.008

[cit0019] Hamid M.A., Sarmidi R.S., Park C.S. (2012) Mangosteen leaf extract increases melanogenesis in B16F1 melanoma cells by stimulating tyrosinase activity in vitro and by up-regulating tyrosinase gene expression. Int. J. Mol. Med. 29: 209–217. 22089762 10.3892/ijmm.2011.840

[cit0020] Halliwell B., Gutteridge J.M.C. (1981) Formation of thiobarbituric acid reactive substances from deoxyribose in the presence of iron salts: the role of superoxide and hydroxyl radicals. FEBS Lett. 128: 347–352. 6266877 10.1016/0014-5793(81)80114-7

[cit0021] Hua Z., Yi-fei Y., Zhi-Qin Z. (2018) Phenolic and flavonoid contents of mandarin (Citrus reticulata Blanco) fruit tissues and their antioxidant capacity as evaluated by DPPH and ABTS methods. J. Integr. Agric. 17: 256–263.

[cit0022] Ines S., Ines B., Wissem B., Mohamed B.S., Nawel H., Dijoux Franca M.G., Kamel G., Leïla C.G. (2012) In vitro antioxidant and antigen otoxic potentials of 3,5 O di galloylquinic acid extracted from Myrtus communis leaves and modulation of cell gene expression by H2O2. J. Appl. Toxicol. 32: 333–341. 21751221 10.1002/jat.1655

[cit0023] Karl J., Samuel P.N., Sornakumar R.S.A. (2020) Antioxidant, antimicrobial, haemolytic, germination and growth promoting properties of Crotalaria juncea L. Plant Sci. Today 7: 201–205.

[cit0024] Khaliq G., Ramzan M. Baloch A.H. (2019) Effect of Aloe vera gel coating enriched with Fagonia indica plant extract on physicochemical and antioxidant activity of sapodilla fruit during postharvest storage. Food Chem. 286: 346–353. 30827617 10.1016/j.foodchem.2019.01.135

[cit0025] Khan R.A., Khan M.R., Sahreen S., Ahmed M. (2012) Evaluation of phenolic contents and antioxidant activity of various solvent extracts of Sonchus asper (L.) Hill. Chem. Central J. 6: 12. 10.1186/1752-153X-6-12 PMC3292812 22305477

[cit0026] Khoshnamvand M., Huo C., Liu J. (2019) Silver nanoparticles synthesized using Allium ampeloprasum L. leaf extract: characterization and performance in catalytic reduction of 4-nitrophenol and antioxidant activity. J. Mol. Struct. 1175: 90–96.

[cit0027] Koolen H.H.F., da Silva F.M.A., Gozzo F.C., de Souza A.Q.L., de Souza A.D.L. (2013) Antioxidant, antimicrobial activities and characterization of phenolic compoundsfrom buriti (Mauritia flexuosa L. f.) by UPLC–ESI-MS/MS. Food Res. Int. 51: 467–473.

[cit0028] Lai X., Wei J., Ding X. (2018) Paeoniflorin antagonizes TNF-α-induced L929 fibroblastoma cells apoptosis by inhibiting NF-κBp65 activation. Dose-Response 16(2): 1559325818774977. 29887769 10.1177/1559325818774977 PMC5989054

[cit0029] Mahmoudi S., Khali M., Benkhaled A., Benamirouche K., Baiti I. (2016) Phenolic and flavonoid contents, antioxidant and antimicrobial activities of leaf extracts from ten Algerian Ficus carica L. varieties. Asian Pac. J. Trop. Biomed. 6: 239–245.

[cit0030] Martino R., Canale F., Sülsen V., Alonso R., Davicino R., Mattar A., Anesini C., Micalizzi B. (2014) A fraction containing kaempferol-3,4-dimethylether from Larrea divaricate Cav. induces macrophage activationon mice infected with Candida albicans. Phytother. Res. 28: 917–924. 24281902 10.1002/ptr.5086

[cit0031] Masoko P., Picard J., Eloff J.N. (2005) Antifungal activities of six South African Terminalia species (Combretaceae). J. Ethnopharmacol. 99: 301–308. 15894142 10.1016/j.jep.2005.01.061

[cit0032] Megdiche-Ksouri W., Trabelsi N., Mkadmini K., Bourgou S., Noumi A., Snoussi M, Barbria R., Tebourbi O., Ksouri R. (2015) Artemisia campestris phenolic compounds have antioxidant and antimicrobial activity. Ind. Crops Prod. 63: 104–113.

[cit0033] Nakilcioglu E., Hısıl Y. (2013) Research on the phenolic compounds in sarilop (Ficus carica L.) fig variety. GIDA 38: 267–274.

[cit0034] Nourbakhsh F., Lotfalizadeh M., Badpeyma M., Shakeri A., Soheili V. (2022) From plants to antimicrobials: natural products against bacterial membranes. Phytother. Res. 36: 33–52. 34532918 10.1002/ptr.7275

[cit0035] Nunes P.X., Silva S.F., Guedes R.J., Almeida S. (2012) Biological oxidations and antioxidant activity of natural products. [in:] *Phytochemicals as Nutraceuticals*. Ed. V. Rao. IntechOpen.

[cit0036] de Oliveira J.R., de Jesus Viegas D., Martins A.P.R., Carvalho C.A.T., Soares C.P., Camargo S.E.A., Jorge A.O.C., de Oliveira L.D. (2017) Thymus vulgaris L. extract has antimicrobial and anti-inflammatory effects in the absence of cytotoxicity and genotoxicity. Arch. Oral. Biol. 82: 271–279. 28683409 10.1016/j.archoralbio.2017.06.031

[cit0037] Olufemi B.E., Olusegun O.V. (2013) Antibacterial properties of ethanolic extract of Ficus carica on microorganisms isolated from pepper Capsicum frutescens. Web. Pub. J. Sci. Res. 1: 7–15.

[cit0038] Pelegrini P.B., Farias L.R., Saude A.C., Costa F.T., Bloch C.J., Silva L.P., Oliveira A.S., Gomes C.E., Sales M.P., Franco O.L. (2009) A novel antimicrobial peptide from Crotalaria pallida seeds with activity against human and phytopathogens. Curr. Microbiol. 59: 400–404. 19641962 10.1007/s00284-009-9451-6

[cit0039] Poljsak B., Šuput D., Milisav I. (2013) Achieving the balance between ROS and antioxidants: when to use the synthetic antioxidants. Oxid. Med. Cell Longev. 2013: 956792. 23738047 10.1155/2013/956792 PMC3657405

[cit0040] Rajeshkumar S., Menon S., Venkat Kumar S., Tambuwala M.M., Bakshi H.A., Mehta M., Satija S., Gupta G., Chellappan D.K., Thangavelu L., Dua K. (2019) Antibacterial and antioxidant potential of biosynthesized copper nano-particles mediated through Cissus arnotiana plant extract. J. Photochem. Photobiol. B 197: 111531. 31212244 10.1016/j.jphotobiol.2019.111531

[cit0041] Rates S.M.K. (2001) Plants as source of drugs. Toxicon 39: 603–613. 11072038 10.1016/s0041-0101(00)00154-9

[cit0042] Saeed N., Khan M.R., Shabbir M. (2012) Antioxidant activity, total phenolic and total flavonoid contents of whole plant extracts Torilis leptophylla L. BMC Compl. Alternat. Med. 12: 221. 10.1186/1472-6882-12-221 PMC3524761 23153304

[cit0043] Şahin S., Elhussein E., Bilgin M., Lorenzo J.M., Barba F.J., Roohinejad S. (2018) Effect of drying method on oleuropein, total phenolic content, flavonoid content, and antioxidant activity of olive (Olea europaea) leaf. J. Food Process Preserv. 42: e13604.

[cit0044] Sahreen S., Khan M.R., Khan R.A. (2011) Phenolic compounds and antioxidant activities of Rumex hastatus D. Don. Leaves. J. Med. Plants Res. 5: 2755–2765.

[cit0045] Sandeep K., Shrivastava B., Khajuria R.K. (2010) Antimicrobial activity of Crotalaria burhia Buch.-Ham. Indian J. Nat. Prod. Resour. 1: 481–484.

[cit0046] Sattayasai J., Chaonapan P., Arkaravichie T., Soi-ampornkul R., Junnu S., Charoensilp P., Samer J., Jantaravinid J., Masaratana P., Suktitipat B., Manissorn J., Thongboonkerd V., Neungton N., Moongkarndi P. (2013) Protective effects of mangosteen extract on H2O2-induced cytotoxicity in SKN-SH cells and scopolamine-induced memory impairment in mice. PLOS One 8: e85053. 24386444 10.1371/journal.pone.0085053 PMC3874002

[cit0047] Smith-Palmer A., Stewart J., Fyfe L. (1998) Antimicrobial properties of plant essential oils and essences against five important food-borne pathogens. Lett. Appl. Microbiol. 26: 118–122. 9569693 10.1046/j.1472-765x.1998.00303.x

[cit0048] Şöhretoğlu D., Sabuncuoğlu S., Harput Ü.Ş. (2012) Evaluation of antioxidative, protective effect against H2O2 induced cytotoxicity, and cytotoxic activities of three different Quercus species. Food Chem. Toxicol. 50: 141–146. 22067294 10.1016/j.fct.2011.10.061

[cit0049] Stefanovic O.D., Tesic J.D., Comic L.R. (2015) Melilotus albus and Dorycnium herbaceum extracts as source of phenolic compounds and their antimicrobial, antibiofilm, and antioxidant potentials. J. Food Drug Anal. 23: 417–424. 28911698 10.1016/j.jfda.2015.01.003 PMC9351790

[cit0050] Tohidi B., Rahimmalek M., Arzani A. (2017) Essential oil composition, total phenolic, flavonoid contents, and antioxidant activity of Thymus species collected from different regions of Iran. Food Chem. 220: 153–161. 27855883 10.1016/j.foodchem.2016.09.203

[cit0051] Tsai W.C., Chang H.C., Yin H.Y., Huang M.C., Agrawal D.C., Wen H.W. (2020) The protective ability and cellular mechanism of Koelreuteria henryi Dummer flower extract against hydrogen peroxide-induced cellular oxidative damage. Electron J. Biotechnol. 47: 89–99.

[cit0052] Turky2lmaz M., Tagı S., Dereli U., Ozkan M. (2013) Effects of various pressing programs and yields on the antioxidant activity, antimicrobial activity, phenolic content and colour of pomegranate juices. Food Chem. 138: 1810–1818. 23411313 10.1016/j.foodchem.2012.11.100

[cit0053] Vanlangenakke N., Bertrand M.J.M., Bogaert P., Vandenabeele P., Berghe T.V. (2011) TNF-ainduced necroptosis in L929 cells is tightly regulated by multiple TNFR1 complex I and II members. Cell Death Dis. 2: e230. 22089168 10.1038/cddis.2011.111 PMC3223695

[cit0054] Yoon Y., Kyung-Seok K., Seong-Gil H., Bong-Joo K., Mi-Young L., Dong-Wuk C. (2000) Protective effects of Orostachys japonicus A. Berger (Crassulaceae) on H2O2-induced apoptosis in GT1-1 mouse hypothalamic neuronal cell line. J. Ethnopharmacol. 69: 73–78. 10661886 10.1016/s0378-8741(99)00107-5

[cit0055] Yuan W., Yuk H.G. (2018) Antimicrobial efficacy of Syzygium antisepticum plant extract against Staphylococcus aureus and methicillin-resistant S. aureus and its application potential with cooked chicken. Food Microbiol. 72: 176–184. 29407395 10.1016/j.fm.2017.12.002

[cit0056] Yusoff N.A., Yam M.F., Beh H.K., Razak K.N.A., Widyawati T., Mahmud R., Ahmad M., Asmawi M.Z. (2015) Antidiabetic and antioxidant activities of Nypa fruticans Wurmb. vinegar sample from Malaysia. Asian Pac. J. Trop. Med. 8: 595–605. 26321511 10.1016/j.apjtm.2015.07.015

[cit0057] Zhao M., Yang Q., Lin L., Sun B., Wang Y. (2017) Intracellular antioxidant activities of selected cereal phenolic extracts and mechanisms underlying the protective effects of adlay phenolic extracts on H2O2-induced oxidative stress in human erythrocytes. J. Funct. Foods 31: 160–171.

